# Reassessment of alcohol consumption in Germany – Which population groups are at increased risk of disease?

**DOI:** 10.25646/13457

**Published:** 2025-09-24

**Authors:** Almut Richter, Anne Starker, Anja Schienkiewitz

**Affiliations:** Robert Koch Institute, Department of Epidemiology and Health Monitoring, Berlin, Germany

**Keywords:** Alcohol consumption, Consumption behaviour, Risk of disease, Health consequences, Adults, Telephone interview, GEDA, EHIS, Germany

## Abstract

**Background:**

According to the new position statement of the German Nutrition Society, there is no level of alcohol consumption that is risk-free to health. One to two alcoholic drinks per week are associated with a low risk of negative health consequences, three to six with a moderate risk, and more than six with a high risk. Alcohol consumption in Germany was assessed according to these categories.

**Methods:**

The analyses are based on the survey German Health Update (GEDA 2019/2020-EHIS) conducted by the Robert Koch Institute with data from 22,708 adults. The frequency and amount of alcohol consumption were asked in a telephone interview.

**Results:**

21.1 % of adults stated that they did not drink alcohol. 46.3 % had an alcohol consumption with a low risk of negative health consequences. 32.5 % of adults reported moderate or high-risk consumption, with significantly more men (44.3 %) than women (21.4 %) doing so. This consumption pattern was most prevalent among men aged 45 to 64 and 65 and over (almost one in two in both groups), as well as among women aged 45 to 64 (about one in four), and increased in both sexes in higher education groups.

**Conclusions:**

Almost one in three adults consume three or more alcoholic drinks per week, which is associated with a moderate to high risk of disease. Therefore, measures proven to reduce alcohol consumption, such as advertising bans, higher taxation and restrictions on availability, should be implemented.

## 1. Introduction

Alcohol consumption is one of the major risk factors worldwide for diseases such as cardiovascular disease, liver disease and cancer, as well as for health impairments and deaths [1]. Alcohol consumption is therefore a major risk factor for premature death and disability, especially in younger age groups [2]. According to data from the Global Burden of Disease (GBD) study, approximately 1.8 million people worldwide died in 2021 due to high alcohol consumption. Of these, approximately 47,500 were in Germany [3].

Alcohol consumption varies considerably around the world [2, 4]. In 2019, Germany was one of the countries with the highest level of alcohol consumption in international comparison, with an average of 12.8 litres of pure alcohol consumed per person aged 15 and over, compared to a global average of 5.8 litres of pure alcohol per person [5].

Due to the negative health consequences of alcohol consumption, recommendations have been made on how to deal with alcohol. According to these recommendations, drinking less than 20 to 24 grams of pure alcohol per day for men and less than 10 to 12 grams of pure alcohol for women was previously considered low risk for healthy people in Germany [6, 7]. 10 grams of pure alcohol corresponds to approximately 0.25 litres of beer or 0.1 litres of wine. However, new findings show that there is no safe level of alcohol consumption [8]. Accordingly, in a position statement published in 2024, the German Nutrition Society (DGE) formulated recommendations for action, advising adults to ‘abstain from alcoholic beverages’. In addition, alcohol consumption levels are assessed in terms of negative health consequences [9]. The risk levels for alcohol consumption mentioned in the DGE position statement were based on calculations by the Canadian Centre on Substance Use and Addiction from 2023 [10], as well as on the results of the GBD study 2020 [11]. The Canadian recommendations were based on a systematic literature review of the short- and long-term consequences of alcohol consumption. Based on this, mathematical modelling was used to determine the amounts of alcohol associated with low risk (defined as one death per 1,000 people) and moderate risk (defined as one death per 100 people). A low risk was determined for one to two standard drinks per week and a moderate risk for up to six standard drinks per week. There was no difference between the sexes. However, pronounced gender differences were found when consumption exceeded six drinks, with the health risks increasing more sharply for women than for men. The GBD study also used updated systematic reviews and meta-regressions to determine the amount of alcohol at which there is no increased health risk (theoretical minimum risk exposure level).

**Figure 1:**
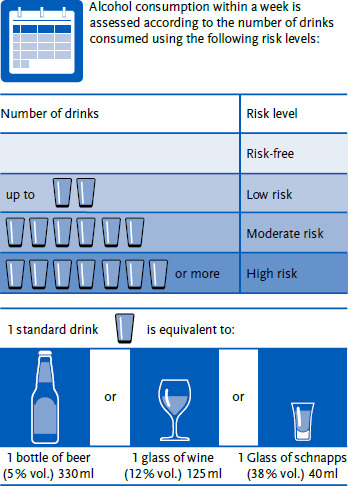
Risk levels of alcohol consumption according to the number of standard drinks. Source: Own figure according to Richter et al. 2024 [9]

In the DGE position statement, the individual risk levels are designated as ‘risk-free’, ‘low risk’, ‘moderate risk’ and ‘risky’. In this article, the risk levels are referred to as risk-free, low risk, moderate risk and high risk, based on the designations used by the Canadian Centre on Substance Use and Addiction in 2023 [10]. These categories are based on the number of alcoholic drinks per week expressed in standard drinks (Figure 1). The drinks shown in Figure 1 are classified as ‘standard drinks’. This means that the risk is low with an alcohol intake of up to 27 grams of pure alcohol per week, moderate with 27 to 81 grams of pure alcohol per week, and high with more than 81 grams of pure alcohol per week.

The amount previously considered low risk was approximately 140 grams of pure alcohol per week for men and 70 grams of pure alcohol per week for women. For women, this old limit now corresponds to amounts that are considered moderately risky. For men, however, the old limit is significantly above the threshold at which consumption is considered high risk according to the new assessment.

**Key messages**

► According to the current state of research, there is no safe level of alcohol consumption.
► Adults should therefore consume no alcohol or as little as possible.
► Consuming three or more alcoholic drinks per week is associated with a moderate to high risk of negative health consequences.
► 21 % of women and more than 44 % of men consume alcohol in quantities that are associated with a moderate or high risk of negative health consequences.
► In addition to raising awareness of the harmful effects of alcohol consumption, restricting alcohol advertising and increasing taxation can help reduce alcohol consumption in Germany.

Regarding the recommendation to abstain from alcohol completely, the DGE’s position statement aligns with the alcohol consumption recommendation by the German Centre for Addiction Issues, the central umbrella organisation for addiction support and self-help in Germany [12].

Previous analyses of alcohol consumption in Germany, based on the old, gender-specific limits, showed that 16.1 % of men and 11.1 % of women had risky alcohol consumption. Particularly among women, there were significant differences between age and education groups [13, 14]. For example, on average, the proportion of women aged 30 to 44 with risky alcohol consumption was lower than in other age groups. In addition, a clear education gradient was observed among women: the proportion with risky consumption increases with the education group. Earlier data, collected as part of health monitoring at the Robert Koch Institute (RKI), also showed differences between genders, age groups and education groups, the latter particularly among women [15, 16].

This article analyses alcohol consumption among the adult population in Germany based on the new risk levels and compares it with previous thresholds. It also examines the sociodemographic characteristics (gender, age, and education) of consumers in the different risk levels.

## 2. Methods

### 2.1 Sample design and study implementation

The German Health Update (GEDA) is a representative cross-sectional survey and part of the nationwide health monitoring at the RKI [17, 18]. The questionnaire from the third wave of the European Health Interview Survey (EHIS) was integrated into the GEDA 2019/2020-EHIS survey. Data collection took place between April 2019 and September 2020 and was conducted as a telephone survey using a computer-assisted, fully structured interview (Computer Assisted Telephone Interview, CATI) [19]. A telephone sample combining mobile and landline numbers (dual-frame method) was used for the survey [20]. A total of 23,001 people aged 15 and over took part. The present analyses took people aged 18 and over into account. Further information on survey design, survey content and response rates are described in Allen et al. [19].

### 2.2 Instruments and indicators

#### Alcohol consumption

The questions on alcohol consumption are part of the EHIS. Study participants were first asked about the frequency of their alcohol consumption in the last twelve months: ‘How often have you had an alcoholic drink, such as beer, wine, sparkling wine, spirits, schnapps, cocktails, mixed alcoholic drinks, liqueurs, homemade or home-distilled alcohol, in the last 12 months?’ They could choose from the following answers: ‘Every day or almost daily’, ‘On 5 – 6 days a week’, ‘On 3 – 4 days a week’, ‘On 1 – 2 days a week’, ‘On 2 – 3 days a month’, ‘Once a month’, ‘Less than once a month’, ‘Not in the last 12 months, as I no longer drink alcohol’, ‘Never, or only a few sips in my whole life’.

**GEDA 2019/2020-EHIS**

Fifth follow-up survey of the German Health Update

**Data holder:** Robert Koch Institute

**Objectives:** Provision of reliable information on the health status, health behaviour and health care of the population living in Germany, with the possibility of European comparisons

**Study design:** Cross-sectional telephone survey

**Population:** German-speaking population aged 15 and older living in private households that can be reached via landline or mobile phone

**Sampling:** Random sample of landline and mobile telephone numbers (dual-frame method) from the ADM sampling system (Arbeitskreis Deutscher Markt- und Sozialforschungsinstitute e.V.)

**Sample size:** 23,001 respondents

**Study period:** April 2019 to September 2020

**GEDA survey waves:** GEDA 2009, GEDA 2010, GEDA 2012, GEDA 2014/2015-EHIS, GEDA 2019/2020-EHIS, GEDA 2021, GEDA Focus, GEDA 2022, GEDA 2023, GEDA 2024

Further information in German is available at

www.rki.de/geda

Those who stated that they drank alcohol at least one to two days per week were then asked on how many days they usually drink alcohol. A distinction was made between weekdays (Monday to Thursday) and weekends (Friday to Sunday). This was followed by a question about the average number of drinks per day of consumption, again separated into weekdays and weekends. Standard drinks were given as examples of a reference quantity for one drink, such as 0.33 litres of beer, a small glass of wine or a glass of sparkling wine, each containing 0.125 litres. The number of drinks was queried in stages: ‘No drinks per day’, ‘1 drink per day’, ‘2 drinks per day’, ‘3 drinks per day’, ‘4 – 5 drinks per day’, ‘6 – 9 drinks per day’, ’10 – 15 drinks per day’, ‘16 or more drinks per day’. The exact wording of the questions (in German) can be found in the GEDA 2019/2020-EHIS questionnaire [21]. This information was used to calculate the average number of alcoholic drinks consumed per week. For the response categories where the number of drinks was asked as a range, the following quantities were used: for 4 to 5 drinks: 4.5; for 6 to 9 drinks: 7.5; for 10 to 15 drinks: 12.5; and for 16 or more drinks: 18.

#### Sociodemographics

Participants were asked about their sex registered at birth and their gender identity [22]. The analysis by gender is based on individuals who identify themselves as female or male. Those who do not assign themselves to these categories (gender-diverse individuals) are not reported separately due to the small number of cases.

To determine participants’ ages at the time of the survey, they were asked for their month and year of birth. For the analysis, participants were divided into four age groups: 18 to 29 years, 30 to 44 years, 45 to 64 years, 65 years and older.

Based on the information provided on education, three education groups were distinguished in accordance with the CASMIN (Comparative Analysis of Social Mobility in Industrial Nations) classification [23]: low (primary or lower secondary education), medium (middle or upper secondary education) and high (higher education).

#### Assessment of alcohol consumption in risk levels

**Figure 2:**
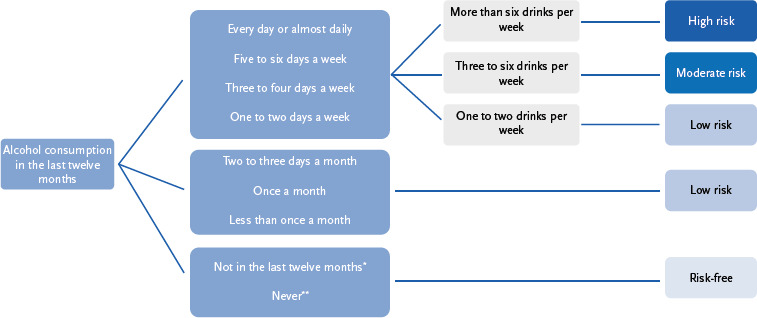
Definition of risk levels of alcohol consumption based on survey data ^*^Response category: Not in the last twelve months, as I no longer drink alcohol. ^**^Response category: Never, or only a few sips or tries, in my whole life.

Alcohol consumption is assessed in four categories: ‘risk-free’, ‘low risk’, ‘moderate risk’ and ‘high risk’. The categories were formed as shown in Figure 2.

### 2.3 Statistical methods

The percentage of the population with 95 % confidence intervals for all four risk levels of alcohol consumption is presented. The results are described for the total population as well as separately for women and men and additionally by age and education groups.

In order to correct for deviations of the sample from the population structure, the analyses were performed using a weighting factor. Weighting was applied for the different selection probabilities (mobile and landline) and an adaptation was made subsequently to the official population figures based on age group, gender, federal state, district type (as of 31 December 2019). In addition, adjustment is made for the education distribution identified by the 2017 Microcensus [19].

The analysis was performed using SAS 9.4. To account for weighting in the calculation of confidence intervals and p-values, all analyses were performed using SAS Survey procedures. The difference between the groups under consideration was assessed as statistically significant if the p-value was less than 0.05 in the Rao-Scott chi-square test, or if the 95 % confidence intervals did not overlap.

## 3. Results

The sample consists of 22,392 individuals aged 18 and older who provided information on their alcohol consumption. A total of 326 participants were excluded due to missing or incomplete information. The sociodemographic characteristics of the study population are shown in Table 1. 54 participants could not be assigned to an education group due to missing information. 60 participants did not identify themselves as either female or male and were not included in the stratified analysis by gender.

Table 2 illustrates the results on alcohol consumption in the population. 25.3 % of women and 16.7 % of men stated that they did not drink alcohol in the last twelve months or do not consume alcohol in general. The latter applies to 16.5 % of women and 10.0 % of men (figures not explicitly shown in Table 2). 53.3 % of women and 39.0 % of men report consuming a maximum of two alcoholic drinks per week, which is considered to pose a low risk of negative health consequences. 12.8 % of women and 15.6 % of men have a moderate risk of negative health consequences with three to six drinks per week, and 8.6 % of women and 28.6 % of men have a high risk with more than six drinks per week. This means that 21.4 % of women and more than twice as many men (44.3 %) consume alcohol at moderate or high-risk levels.

**Figure 3:**
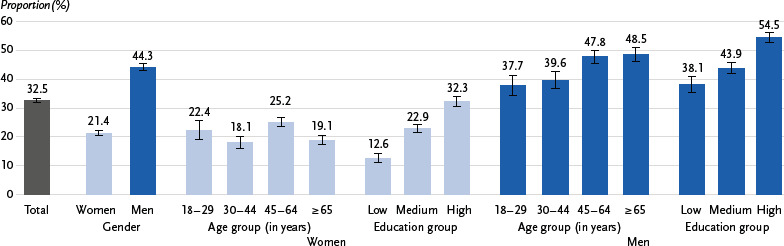
Proportion of the population with moderate or high-risk alcohol consumption by gender, age and education (n = 11,817 women, n = 10,515 men). Source: GEDA 2019/2020-EHIS

The group with moderate or high-risk alcohol consumption is described separately for women and men below. For this consumption pattern, there are statistically significant differences between age and education groups for both sexes (p < 0.0001 in each case). Almost half of men aged 45 to 64 (47.8 %) and men aged 65 and over (48.5 %) consume moderate or high-risk amounts of alcohol (Figure 3). In these age groups, the proportion of consumers at high risk of negative health consequences is also high (Table 2).

Among women aged 45 to 64, a higher proportion of those who drink alcohol at moderate or high risk are observed compared to the adjacent younger and older groups (Figure 3). On a positive note, the proportion of women with high-risk alcohol consumption is particularly low among those aged 30 to 44 (6.6 %; Table 2).

In addition, a correlation with education is evident for both sexes: the higher the education group, the higher the proportion of those who consume alcohol at a moderate or high-risk level. Among men in the high education group, this applies to more than half, while among women in the high education group, it applies to almost one in three (Figure 3).

###  

#### Comparison of the results with the previous definition of risky alcohol consumption

The DGE position statement clearly states that people should consume as little alcohol as possible. Consuming one to two alcoholic drinks per week is associated with a low risk of negative health consequences [9]. This is significantly below what has previously been considered as low-risk consumption (a maximum of one glass per day for women and a maximum of two glasses per day for men) [6, 7]. Due to this new risk definition, a significantly larger proportion of the population has an alcohol consumption above a low risk level. Whereas it was previously assumed that 13.5 % of the population was affected [13], the new definition yields a figure of 32.5 % based on the same data. The difference is particularly large among men: previously, 16.1 % of men were assumed to have a consumption above the low-risk level [14]. According to the new definition, 44.3 % of men drink alcohol in quantities that exceed a low risk of negative health consequences. Among women, the proportion with consumption above low risk was previously 11.1 % [14] and is now 21.4 % with consumption above low risk, which corresponds to a doubling of the proportions.

With a low overall proportion of men exceeding the old threshold for risky consumption (16.1 %), there were hardly any differences between age and education groups to date. However, the new risk levels make it clear that men in the age groups 45 to 64 and 65 and older in particular consume alcohol in quantities above the low-risk level. As with women, there is now also a clear educational gradient to the detriment of the higher education groups among men.

## 4. Discussion

### 4.1 Main results

In Germany, 78.8 % of adults consume alcoholic beverages, 74.7 % of women and 83.3 % of men. The reassessment of alcohol consumption based on the DGE position statement published in 2024 shows that 21.4 % of women and 44.3 % of men consume alcohol in quantities associated with a moderate or high risk of negative health effects. The proportion of people with moderate or high-risk alcohol consumption is particularly high in the age group 45 to 64 and, among men, also in the age group 65 and older. This also applies to people in the high education group: more than half of men and almost one in three women with a high level of education drink alcohol in quantities associated with a moderate or high risk of negative health consequences. Following the reassessment, the proportion of people whose alcohol consumption is classified as above the low risk threshold has risen from 16.1 % to 44.3 % for men and from 11.1 % to 21.4 % for women, when comparing the old definition with the new definition. Any health risk associated with alcohol can only be avoided by abstaining completely. This is currently not being implemented by the majority of the population.

### 4.2 Contextualisation and interpretation

There are various epidemiological studies in Germany that record alcohol consumption among the population, e.g. the Epidemiological Survey of Substance Abuse (ESA). In addition, official statistics record key figures on alcohol consumption, e.g. alcohol tax statistics. The different focuses and methodological approaches of these studies and statistics enable a differentiated assessment of alcohol consumption and its consequences in the population. However, this results in key figures that are not comparable and may differ due to different survey methods, periods, and the application of different thresholds and definitions. Overall, the per capita consumption of pure alcohol in Germany has declined slightly in recent decades [24].

For the first time in Germany, an assessment of alcohol consumption among the adult population has been carried out according to the risk levels outlined in the current DGE position statement, using the available data. A direct comparison with the results of other studies or statistics is not possible due to the different risk levels. Only the proportion of people who are abstinent is comparable. In our sample, 21.2 % of respondents stated that they had been abstinent throughout their lives or at least in the last twelve months. In 2021, the ESA found that 4.9 % of 18- to 64-year-olds had never consumed alcohol and 11.2 % had not consumed alcohol in the last twelve months; at 16.1 % overall, this is a slightly lower proportion than in our survey [25]. However, the different age groups make comparison difficult, as unlike the ESA, there is no upper age limit in GEDA 2019/2020-EHIS.

The new risk levels, with a higher risk of negative health consequences as consumption increases, illustrate the dose-response relationship better than the previous threshold. In addition, communicating potential health risks is made easier by translating alcohol intake into specific numbers of drinks, and by the risk levels now being gender-neutral. However, the key recommendation remains that any alcohol consumption is associated with risks.

For this reason, the German Centre for Addiction Issues no longer specifies limit values and generally recommends consuming no alcohol or as little as possible [12]. This is also explained by differences between individuals, e.g. in terms of age or genetic factors, as well as in relation to vulnerable population groups, including children, adolescents, pregnant women, breastfeeding women, people with pre-existing conditions or those taking medication.

Binge drinking, defined as consuming six or more alcoholic drinks on a single occasion, is a particularly risky drinking behaviour in terms of health. High amounts of alcohol and binge drinking should be avoided in all cases [9].

### 4.3 Strengths and limitations

The present analyses are based on data from a representative sample of the adult population in Germany. The firsttime application of the new risk levels for alcohol consumption in accordance with the DGE position statement enables an assessment of reported alcohol consumption across the entire population, based on the latest findings, and allows for differentiation according to sociodemographic characteristics.

However, the study design and data collection methods give rise to various limitations. Estimates of alcohol consumption based on self-reported drinking frequency and quantities may be inaccurate. For example, the possible influence of recall bias on alcohol consumption in the last twelve months cannot be ruled out. Similarly, possible fluctuations in usual drinking frequency and average drinking quantities are not taken into account. The number of drinks is partly recorded using ranges. The quantities of alcohol consumed are based on standard drinks and serve as a guide; the actual amount consumed may differ. As a result, alcohol consumption may be under- or overestimated. However, the questions are based on the established EHIS instrument [26], which is used throughout Europe and enables a European comparison [27].

**Infobox Web portal of Federal Health Reporting**

The website www.gbe.rki.de/EN of Federal Health Reporting at the Robert Koch Institute (RKI) provides reliable information on the health situation of the population in Germany: timely, transparent and easily accessible. The focus is on noncommunicable diseases such as diabetes mellitus, cardiovascular diseases, cancer and mental disorders. It also presents factors that have an influence on health, such as health behaviour or social determinants. In addition, the web portal provides information on health care and contextual factors, such as food taxation or tobacco control measures, which also influence the health of the population.

The website currently includes over 60 indicators from health monitoring at the RKI and other data sources, which are interactively visualised and contextualised in short texts. The data is published as open data on GitHub and Zenodo. In addition, the website provides access to all RKI publications that are related to the topics on the website. The content is continuously expanded.

Further information on the topic of this article can be found on the web portal at

www.gbe.rki.de/alcohol-consumption-risk-levels

Furthermore, the results may have been influenced by socially desirable responses from participants regarding the amount of alcohol consumed [28]. Telephone interviews are more susceptible to this than face-to-face interviews [29]. As a result, alcohol consumption may be underestimated. Telephone surveys also carry the risk of non-response bias, whereby respondents differ systematically from those who refused to participate. It can therefore be assumed that people who participate in a health study also have a higher health awareness and may differ from the general population in terms of their alcohol consumption, for example. It is also conceivable that people with serious health problems were not adequately represented in the study. In addition, people in the low education group were less likely to participate. A weighting factor was applied to reduce this possible selection effect. The survey period between April 2019 and September 2020 also includes the start of the COVID-19 pandemic in March 2020, which may have had an impact on alcohol consumption [30, 31] and therefore does not represent the usual consumption for some of the participants. However, the reference period for alcohol consumption is the last twelve months, so short-term fluctuations are less significant here than in shorter survey periods.

One limitation of calculations regarding the harmfulness of alcohol is that it cannot be ruled out that the studies on the short- and long-term consequences of alcohol consumption included in the systematic literature reviews may also have classified individuals as non-consumers who have stopped drinking due to health problems (‘ill quitters’). This may lead to a bias in which the risks of low-, moderate- and high-risk consumption groups are underestimated in comparison to non-consumers [32].

### 4.4 Recommendations for action

The reassessment of alcohol consumption in Germany based on the current DGE position statement highlights that health risks due to alcohol consumption are widespread across the population. This clearly shows the need for effective preventive measures to minimise health risks. The World Health Organization recommends various measures to reduce alcohol consumption and its consequences. These include restrictions on the availability of alcoholic beverages; measures against drink-driving; facilitating access to preventive medical check-ups, brief interventions and treatments; enforcing bans or comprehensive restrictions on alcohol advertising, sponsorship and promotion; and increasing the price of alcohol through excise duties and pricing policies [33]. A policy mix that involves a variety of actors and consists of a combination of environmental and behavioural preventive measures is considered promising [1].

A recent assessment of the implementation of these measures in Germany concludes that, above all, access to alcohol should be restricted and excise duties on alcoholic beverages should be significantly increased or introduced for wine [24]. Currently, alcoholic beverages can be purchased at any time in Germany and the price is comparatively low: compared to other European countries, Germany ranks third from last in terms of alcohol tax rates [34]. In addition, the legal minimum age for purchasing alcoholic beverages in Germany is low at 16 years; raising it could reduce alcohol consumption among young people and the associated risks [35].

In the area of contextual prevention, raising political and social awareness of the negative effects of alcohol consumption is a priority [1] and an important prerequisite for the effective implementation of the public health goal of ‘Reducing alcohol consumption’ [36]. It states that ‘an important component of alcohol prevention in a well-informed society is the comprehensive, continuous and attention-grabbing provision of information to the population about low-risk alcohol consumption. Providing information in this way is also linked to the goal of keeping the topic of ‘alcohol prevention’ on the political agenda and ensuring that it is the subject of political action’ [36]. The new recommendations for action on alcohol consumption are also intended to serve this purpose.

### 4.5 Conclusion

This article re-evaluates alcohol consumption among the adult population in Germany based on the risk levels outlined in the position statement published by the German Nutrition Society. Accordingly, a significant proportion of the population consumes alcohol at levels that are harmful to their health. The results highlight the urgent need for measures to reduce alcohol consumption across the population. Targeted health policy action is necessary, and the World Health Organization has presented concrete recommendations for this. In addition, education about the negative consequences of alcohol consumption is an important part of prevention, as is the broad communication of the recommendation to drink as little alcohol as possible or none at all.

## Figures and Tables

**Table 1:** Description of the study population (n = 22,392). Source: GEDA 2019/2020-EHIS

	Number	Unweighted	Weighted
		%	%
**Gender identity**			
Female	11,817	52.8	51.1
Male	10,515	47.0	48.5
Diverse	60	0.3	0.4
**Age group (in years)**			
18 – 29	2,089	9.3	16.4
30 – 44	3,741	16.7	22.7
45 – 64	8,878	39.7	35.1
≥ 65	7,684	34.3	25.8
Education group^*^			
Low	4,202	18.8	29.6
Medium	9,843	44.1	52.3
High	8,293	37.1	18.0

^*^Missing data for education groups: n = 54

**Table 2:** Proportion of the population by amount of alcohol consumption in risk levels by gender, age and education (n = 11,817 women, n = 10,515 men). Source: GEDA 2019/2020-EHIS

	Number	Risk-free (no alcohol consumption)		Low risk (one to two drinks per week)		Moderate risk (three to six drinks per week)		High risk (seven or more drinks per week)	
		%	(95 % CI)	%	(95 % CI)	%	(95 % CI)	%	(95 % CI)
**Total**	22,392	21.2	(20.3 – 22.1)	46.3	(45.3 – 47.3)	14.2	(13.6 – 14.8)	18.3	(17.6 – 19.1)
**Gender**									
Women	11,817	25.3	(24.0 – 26.6)	53.3	(51.9 – 54.7)	12.8	(12.0 – 13.7)	8.6	(7.9 – 9.3)
Men	10,515	16.7	(15.6 – 17.9)	39.0	(37.6 – 40.5)	15.6	(14.7 – 16.6)	28.6	(27.3 – 29.9)
**Women**									
**Age group (in years)**									
18 – 29	896	18.6	(15.4 – 22.3)	59.0	(54.7 – 63.1)	11.4	(9.1 – 14.2)	11.0	(8.5 – 14.0)
30 – 44	1,885	22.9	(20.2 – 25.9)	59.0	(55.9 – 62.1)	11.4	(9.7 – 13.4)	6.6	(5.4 – 8.1)
45 – 64	4,790	21.8	(19.9 – 23.8)	53.0	(50.9 – 55.1)	16.1	(14.7 – 17.6)	9.1	(8.1 – 10.3)
≥ 65	4,246	34.8	(32.4 – 37.2)	46.2	(43.8 – 48.6)	10.8	(9.6 – 12.1)	8.3	(7.2 – 9.5)
**Education group**									
Low	2,249	41.8	(38.9 – 44.7)	45.6	(42.8 – 48.5)	6.9	(5.7 – 8.4)	5.6	(4.5 – 7.0)
Medium	5,653	20.0	(18.5 – 21.6)	57.1	(55.3 – 59.0)	13.8	(12.6 – 15.1)	9.1	(8.1 – 10.2)
High	3,888	14.3	(12.8 – 15.8)	53.4	(51.3 – 55.6)	20.1	(18.5 – 21.8)	12.3	(10.9 – 13.7)
**Men**									
**Age group (in years)**									
18 – 29	1,180	15.3	(12.7 – 18.4)	47.0	(43.3 – 50.7)	11.7	(9.5 – 14.3)	26.1	(22.9 – 29.5)
30 – 44	1,846	13.7	(11.4 – 16.3)	46.7	(43.4 – 50.0)	15.6	(13.6 – 17.8)	24.1	(21.4 – 27.0)
45 – 64	4,070	17.9	(15.9 – 20.1)	34.3	(32.1 – 36.6)	17.5	(16.0 – 19.1)	30.3	(28.2 – 32.5)
≥ 65	3,419	19.0	(16.9 – 21.3)	32.5	(30.1 – 35.1)	16.0	(14.2 – 17.9)	32.5	(30.0 – 35.1)
**Education group**									
Low	1,938	24.6	(22.0 – 27.5)	37.3	(34.3 – 40.4)	12.2	(10.5 – 14.2)	25.8	(23.2 – 28.7)
Medium	4,166	15.2	(13.6 – 16.8)	40.9	(38.9 – 43.0)	15.2	(13.8 – 16.7)	28.7	(26.8 – 30.6)
High	4,384	8.5	(7.5 – 9.6)	37.1	(35.2 – 39.0)	22.1	(20.6 – 23.7)	32.4	(30.6 – 34.2)

CI = confidence interval
